# P-870. Clinician Alert Interactions and Antibiotic Exposure in Beta-Lactam Allergic Patients following Suppression of Beta Lactam Antibiotic (BLA) Cross-Sensitivity Alerts (CSAs) in the Electronic Health Record (EHR): A Need for Balancing Measures

**DOI:** 10.1093/ofid/ofaf695.1078

**Published:** 2026-01-11

**Authors:** Justin Foster, Katelyn Quartuccio, Stephanie Shulder, Sarah L Spitznogle, David Dobrzynski, Jessica Stern, Alexandra Yamshchikov

**Affiliations:** URMC, Rochester, New York; University of Rochester Medical Center, Highland Hospital, Rochester, NY; University of Rochester Medical Center, Rochester, NY; University of Rochester Medical Center, Rochester, NY; University of Rochester Medical Center, Rochester, NY; Univeristy of Rochester Medical Center, Rochester, New York; University of Rochester School of Medicine and Dentistry, Rochester, NY

## Abstract

**Background:**

Allergies to BLA may affect ∼10% of the population, few are IgE-mediated, and cross-reactivity between agents is low. Removing CSAs from EHRs may increase BLA use. Impact of such interventions on prescribing behavior, interactions with EHR alerts, and antibiotic administration patterns is poorly understood.

**Table 1.**
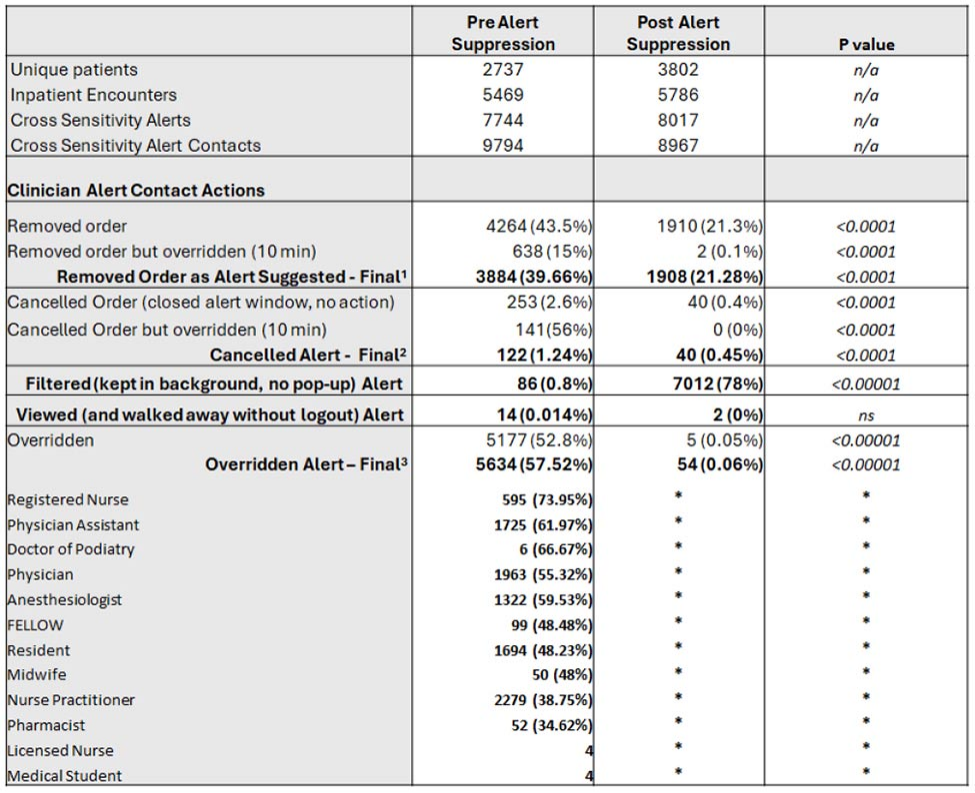
Characterization of Beta Lactam Cross-Sensitivity Alerts and Clinician Actions to Alert Contacts

**Table 2.**
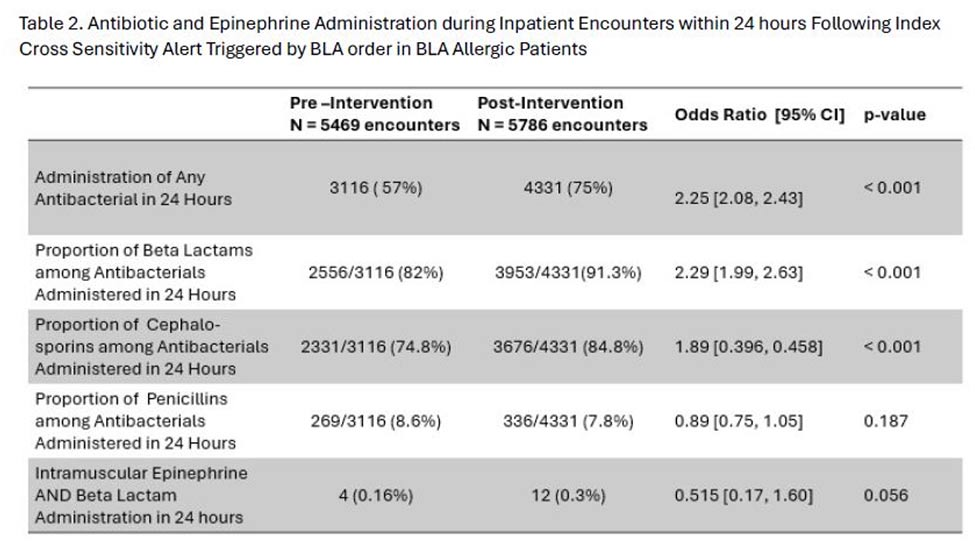
Antibiotic and Epinephrine Administration during Inpatient Encounters within 24 Hours Following Index Cross Sensitivity Alert Triggered by Beta-Lactam Orders in Beta Lactam Allergic Patients

**Methods:**

A quality improvement (QI) intervention consisting of suppression of CSAs for BLA orders within our Epic Systems™ EHR, clinician education, guideline and order set updates was undertaken at a large academic hospital. CSA contacts by clinicians (excluding verifying pharmacist and one step intraoperative anesthesiologist orders) during an index inpatient BLA order for BLA allergic patients pre- (10/1/2022 -3/31/23) and post- (10/1/2023 – 3/31/2024) were reviewed. Confounding alerts for multiple beta-lactam allergies were excluded, no exclusions were made for allergy type, severity, or treatment indication. Actions taken in response to the alert or lack thereof were compared pre and post intervention (Table 1). Systemic antibacterial administrations and intramuscular (IM) epinephrine within 24 hours of alert contact were evaluated. Statistical analysis was conducted using chi square and fisher’s exact test in GraphPad Prism version 2025.

**Results:**

Total 18761 CSA contacts were examined, encompassing 11255 inpatient encounters for 6539 unique patients. Baseline overall alert override rate was variable relative to clinician role, with an average of 57.52% override expectedly decreasing to 0.06% (p < 0.00001) postintervention (Table 1). Inpatient encounters for BLA allergic patients triggering an index CSA via a BLA order were more likely to incur documented administration of any antibacterial (57% vs 75%, P< 0.001) with higher proportion of beta lactams (82% vs 91.3%, P< 0.001). There was no difference in IM epinephrine administrations (Table 2).

**Conclusion:**

A comprehensive QI intervention focused on suppression of frequently overridden BLA CSAs enhanced likelihood of allergic patients receiving BLA at index inpatient antibiotic prescribing, without an increase in severe immediate anaphylactic reactions requiring epinephrine. More studies are needed to evaluate the impact of such interventions on stewardship goals in BLA allergic patients.

**Disclosures:**

David Dobrzynski, Jr., MD, DynaMed: Advisor/Consultant|Innoviva Therapeutics Inc: Advisor/Consultant

